# Is a high-risk clinical target volume required? Evaluation of the dosimetric feasibility based on T staging

**DOI:** 10.3389/fonc.2022.800400

**Published:** 2022-09-02

**Authors:** Xingxing Yuan, Chao Yan, Shiyi Peng, Zhiping Chen, Tianzhu Lu, Qiaoying Gong, Yang Qiu, Wenming Xiong, Fenghua Ao, Guoqing Li, Jingao Li, Ziwei Tu

**Affiliations:** ^1^ National Health Commission (NHC) Key Laboratory of Personalized Diagnosis and Treatment of Nasopharyngeal Carcinoma (Jiangxi Cancer Hospital of Nanchang University), Nanchang, China; ^2^ Department of Radiation Oncology, Jiangxi Cancer Hospital, Nanchang, China

**Keywords:** IMRT, nasopharyngeal carcinoma, omitting CTVp1, plan evaluation, T staging

## Abstract

**Background:**

Clinical target delineation is a primary focus in the field of radiotherapy. This study aimed to investigate whether high-risk clinical target volume can be removed in nasopharyngeal carcinoma patients with different T stages.

**Materials and methods:**

We designed a test plan without the high-risk clinical target volume for 111 nasopharyngeal carcinoma patients and further compared the test plans with the treatment plans in the parameters of planning target volumes and the radiation dose to normal organs.

**Results:**

Our data showed that when high-risk clinical target volume was abnegated, target coverage, conformity indices, and homogeneity indices of planning target volumes and doses of normal organs were not influenced in the T4 nasopharyngeal carcinoma patients, and more than 95% of the high-risk planning target volume area could still be covered by the 60 Gy dose line. However, only some T1–3 patients achieved the ideal dose coverage, and even fewer after induction chemotherapy (62.8% vs. 41.2%, *p* = 0.018). Gross tumor volume was positively correlated with the target coverage of the original high-risk planning target volume in the test-plan (*p* = 0.0001). Gross tumor volume can be used to predict whether the target coverage of high-risk planning target volume is more than 95% (area under the curve = 0.868).

**Conclusion:**

Omitting high risk clinical target volume can be considered in patients with T4 nasopharyngeal carcinoma according to physical evaluations. However, this approach is only suitable for a specific subset of T1–3 patients.

## Introduction

Radiotherapy is still considered the cornerstone of comprehensive treatment for patients with nasopharyngeal carcinoma (NPC) (1), although studies have confirmed that chemotherapy, targeted therapy, and immunotherapy may also improve prognoses of patients with NPC (1, 2). Intensity-modulated radiation therapy (IMRT) has gradually replaced two-dimensional radiotherapy and three-dimensional conformal radiotherapy as the main radiotherapy technique worldwide because of reduced radiation-induced toxicity and improved overall survival of patients (3, 4). However, the advantage of IMRT depends on accurate radiotherapy planning, in which target delineation plays a dominant role (5).

Despite the publication of consensus guidelines based on expert opinion in 2018 (6), in practice, it remains uncertain how far gross tumor volume (GTVp) should be expanded to clinical target volume (CTVp) and whether the whole nasopharyngeal mucosa should be included in high-risk clinical target volume(CTVp1). It is understandable that during target mapping, the enlarged CTVp of advanced patients (stage T3 or 4) with a large tumor often requires modification due to dose limitations of adjacent healthy organs. In other words, the size of the tumor can affect the area of the enlarged CTVp. It has been reported that the higher the T stage, the smaller the average distance from GTVp to CTVp; moreover, the local control rate of patients with different extension distances is similar (7).

Lin et al. proposed the reduced-volume IMRT technique, which omits the contouring of CTVp1 and expands only 8 mm from the gross tumor volume of primary nasopharyngeal lesions and positive retropharyngeal lymph nodes (GTVnx) to low-risk clinical target volume (CTVp2). Dosimetric evaluation showed that the target area of CTVp1 was well covered by the 60 Gy isodose curve. This approach is further supported by excellent local control and survival rates (8). However, the CTVp2 delineation method used in this study is not a mainstream approach in the clinic; thus, whether the abandonment of CTVp1 can be extended into other centers remains to be seen.

To determine whether CTVp1 can be removed according to conventional target area delineation criteria, we developed new plans for NPC patients following radiotherapy based on the contouring method of removing CTVp1 and compared treatment and newly designed radiotherapy plans by physical evaluation.

## Materials and methods

### Patients and treatment

From March 2015 to May 2020, 111 patients with newly treated non-metastatic NPC at Jiangxi Cancer Hospital were enrolled. Patients underwent radiotherapy or chemoradiotherapy according to National Comprehensive Cancer Network guidelines, and all patients received IMRT.

There are subtle differences in the delineation of the target area between clinicians; nevertheless, the general principle is largely consistent among centers (6, 9–11). GTVp included primary nasopharyngeal lesions, positive retropharyngeal lymph nodes (GTVnx), and cervical lymph nodes (GTVnd). High-risk CTVs (CTVp1) were formed by a 5–10-mm expansion of the GTVnx, with or without the entire nasopharyngeal mucosa, and a 5-mm submucosa was sketched. Low-risk CTVs (CTVp2) were diagnosed as malignant based on imaging and clinical evaluation, which included a margin of 5–10 mm to CTVp1 and structures as follow: the posterior nasal cavity, parapharyngeal space, skull base, pterygoid fossa, inferior sphenoid sinus, anterior clivus, elective neck area from level IB to level V, and other structures based on the T and N stages. Planning target volumes of GTVnx, GTVnd, CTVp1 and CTVp2 (PTVnx, PTVnd, PTV1, PTV2) were generated by an expansion of 3–5 mm separately. Adjacent organs at risk included the temporal lobe, brain stem, spinal cord, optic nerves, optic chiasm, lens, parotid glands, and temporomandibular joints. Prescribed doses were 66–70 Gy to GTVnx and GTVnd, 60 Gy to CTV1, and 54 Gy to CTV2 in 30–33 fractions.

### Plan evaluation

The patient’s treatment plan was named as the treat-plan. We removed the dose limitation of PTV1 for each treatment plan and redesigned a corresponding test plan. The test-plans were physically compared to treat-plans, but not used for radiotherapy. Dose-volume histograms were generated for the plan evaluation. All plans fulfilled the following criteria: 1) at least 95% of the target volume received the prescribed dose; 2) no greater than 1% of GTVp was outside the 93% of the prescribed dose range; 3) the maximum point dose was within the GTVp; 4) less than 15% of the PTV received > 110% of the prescribed dose. The doses limitations of critical organs at risk (OARs) were the same for the treatment and test-plans. In addition, the following parameters were calculated to evaluate the treatment and test plans. Conformity Index (CI) was defined as a ratio of prescription isodose coverage volume (V_pres_) to PTV volume (VT): CI = V_pres_/VT. Target Coverage (TC) was the target volume receiving at least the prescription dose (VTpres) divided by the entire target volume (VT): TC = VT_pres_/VT. When TC equaled 1, the target was completely covered. Homogeneity Index (HI) indicated dose homogeneity in the target volumes and referred to the ratio of the dose difference between the greatest dose delivered to 2% of the target volume (D_2%_) and the dose delivered to 98% of the target volume (D_98%_) to the target median dose (D_median_): HI = (D_2%−_D_98%_)/D_median_ (12).

### Statistical analysis

Statistical analysis was performed using SPSS version 26 and GraphPad Prism 8. Plan evaluation data are expressed as means and standard deviations. A t-test was used to compare TC between the induced chemotherapy (IC) and non-IC groups. Pearson correlation analysis was used to evaluate the correlation between GTV and TC. A receiver operating characteristic (ROC) curve was used to investigate the prediction efficiency of GTV for whether the TC of the original PTV1 region reached 95% or higher when CTV1 was not delineated.

## Results

### Patient characteristics and treatment data

A total of 111 NPC patients were included in this study, and clinical characteristics are presented in **Table 1**. The median age of patients was 50 years (range 22–77 years), and 70.3% were men. The T stage distribution was T1–3 in 52 cases and T4 in 59 cases. Forty-three patients received induced radiotherapy, and 68 patients did not.

**Table 1:** Clinical characteristics of patients.

Variables		Cases	Proportion (%)
Age	≤45	32	28.8
	>45	79	71.2
Sex	Male	78	70.3
	Female	33	29.7
T stage	1-3	52	46.8
	4	59	53.2
N stage	0-1	56	50.5
	2-3	55	49.5
Clinical stage	I-II	14	12.6
	III-IV	97	87.4
Treatment	IC*	43	38.7
	Non-IC	68	61.3

*IC, Induced chemotherapy.

### PTV1 evaluation of the test plan

**Figure 1:**
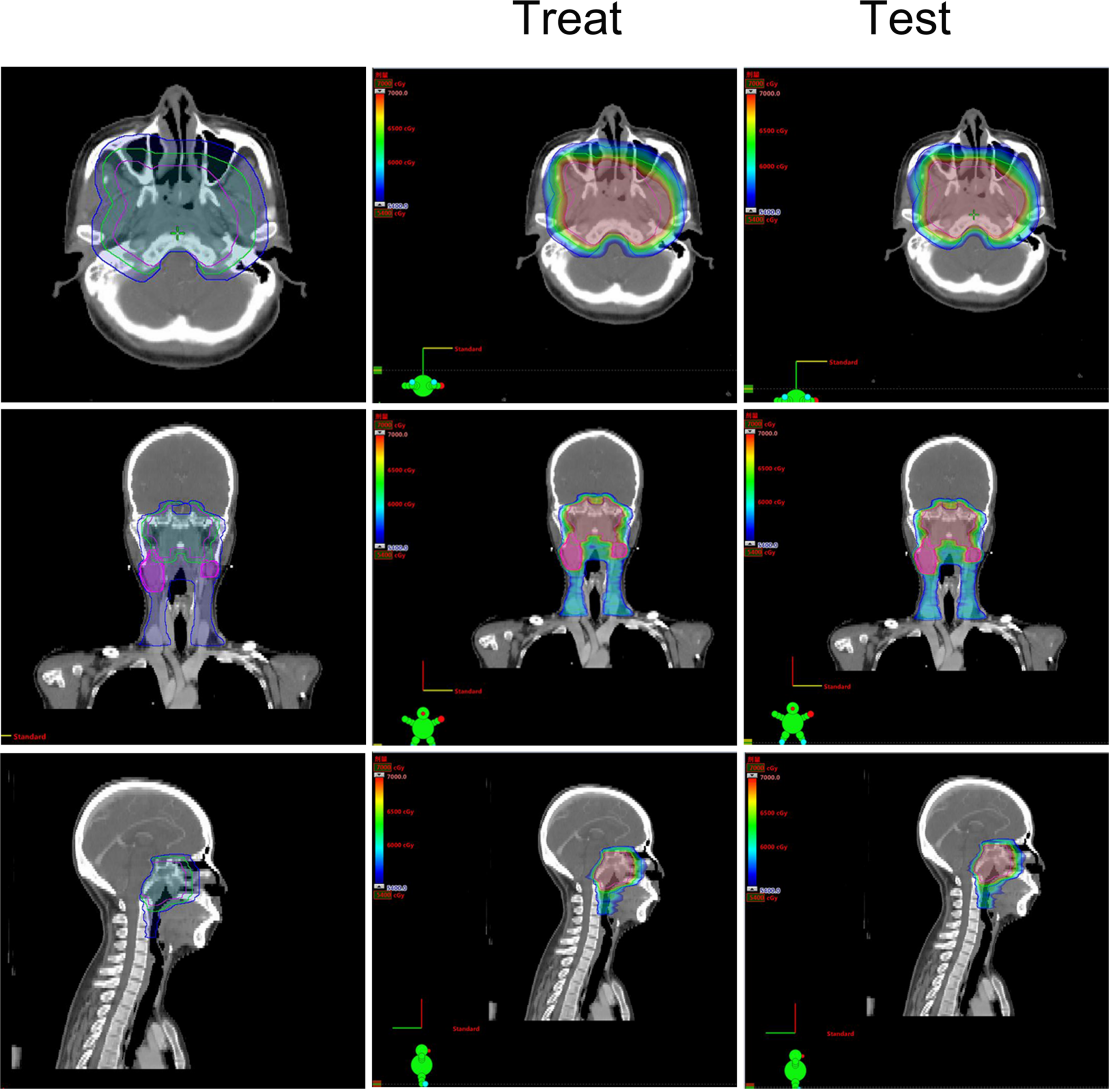
The treatment target area and dose distribution of a T4 nasopharyngeal carcinoma patient. Left: The treatment target area diagram; Middle: The dose distribution diagram of treat-plan; Right: The dose distribution diagram of test-plan.

**Figure 2:**
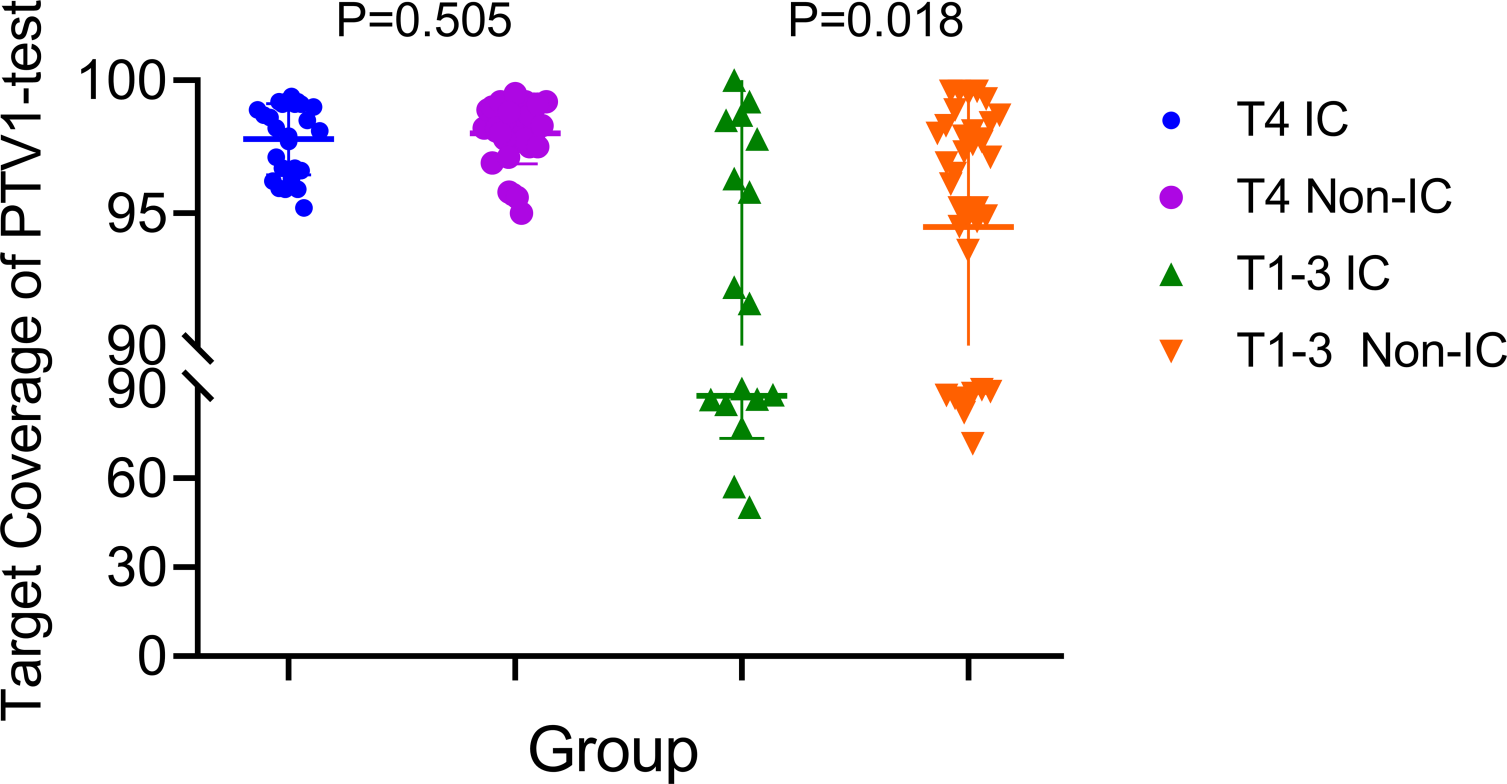
The target coverage of PTV1 in T4 and T1–3patients nasopharyngeal carcinoma patients received induction chemotherapy or not.

Based on the existing treat-plan, a new CTV1 elimination plan was developed as a test plan for each patient, and a total of 222 plans were analyzed. The dose profiles of the treatment and test plans were similar, as shown in **Figure 1**. To determine whether CTV1 can be removed, we first assessed whether the TC of PTV1 in the test plan exceeded 95%. Given that the delineation of the target area following induced chemotherapy (IC) remains controversial, we analyzed the TC of the IC and non-IC groups separately. Our data (**Table 2** and **Figure 2**) showed that in T4 patients, regardless of whether or not patients received IC before radiotherapy, the TC of PTV1 was above 95%. However, only partial T1–3 patients reached 95% or more, and the proportion of whom was significantly reduced after induced chemotherapy. (62.8% vs. 41.2%, *p* = 0.018).

**Table 2:** Target Coverage of PTV1-test.

T stage	Group	Cases rates of TC ≥ 95%
T1-3	IC*	41.2%
	Non-IC	62.8%
T4	IC	100%
	Non-IC	100%

*IC, Induced chemotherapy.

**Figure 3:**
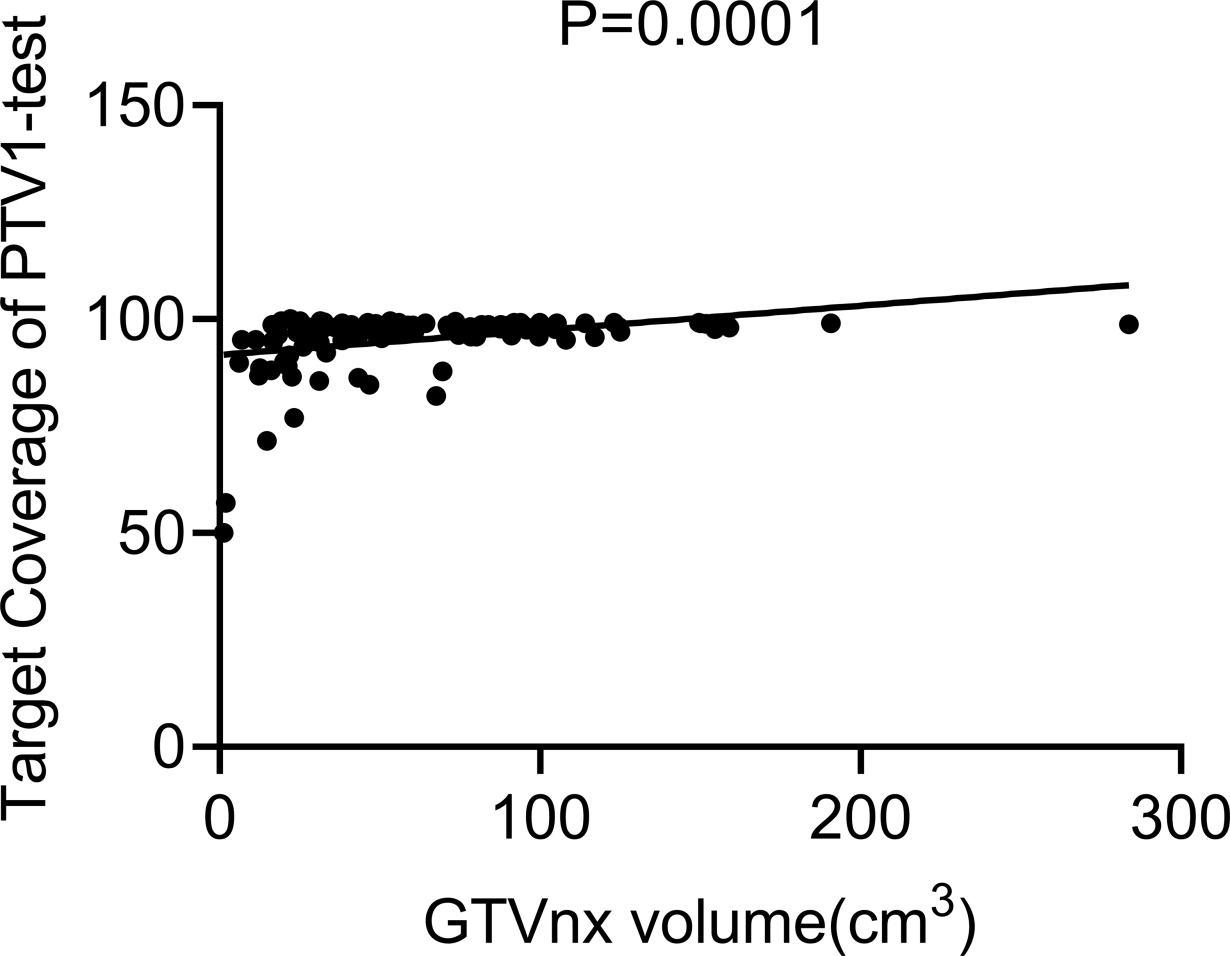
Correlation analysis chart between GTVnx volume and the target coverage of PTV1.

**Figure 4:**
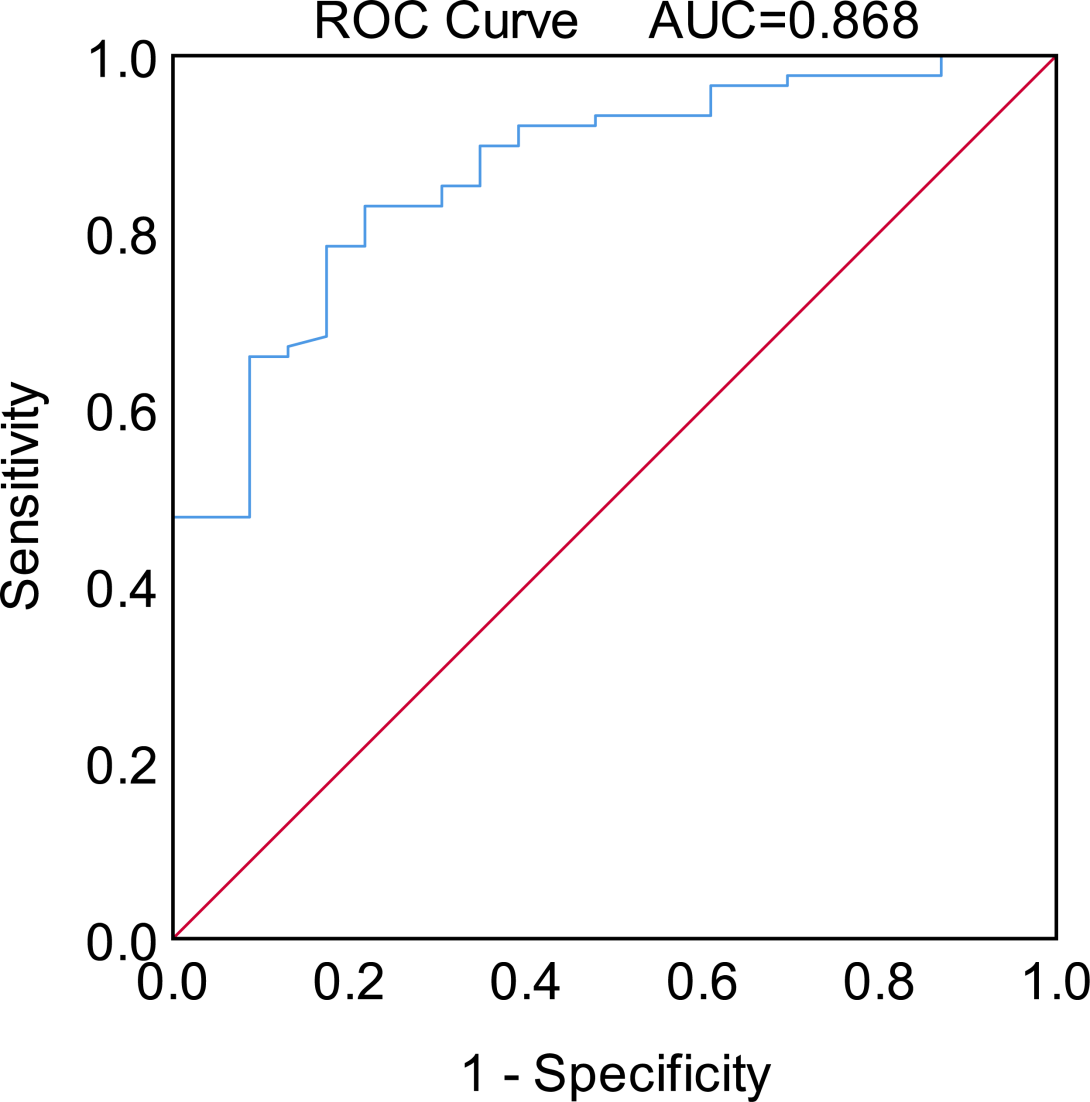
Receiver operating characteristic (ROC) curves for the predictive value of using GTVnx volume to discriminate whether the prescribed dose (60 Gy) cover at least 95% of the volume of PTV1.

The Pearson correlation analysis showed that the GTV was positively correlated with TC of the original PTV1 region in the test plan (*p* = 0.0001, **Figure 3**). The ROC curve showed that GTV could predict whether the PTV1-TC of the CTVp1-omitting test plan was more than 95% (area under the curve [AUC] = 0.868, **Figure 4**).

### Comparison between the two IMRT plans in T4 patients

To verify the feasibility of eliminating CTV1 in T4 NPC patients, we further evaluated the parameters of other target areas and normal organs. Results showed that the TC, CI, and HI of PTVnx, PTVnd, and PTV2 of the test plan were comparable to those of the treatment plan (**Table 3**). Moreover, the radiation doses of the brainstem, spinal cord, optic chiasma, and temporal lobe were similar across the two plans (**Table 4**).

**Table 3:** Target area evaluation in T4 patients.

		TC	CI	HI
PTVnx	Treat-plan	97.331 ± 1.025	1.125 ± 0.095	0.058 ± 0.015
	Test-plan	97.184 ± 1.023	1.125 ± 0.092	0.063 ± 0.017
PTVnd	Treat-plan	97.424 ± 1.186	1.209 ± 0.119	0.063 ± 0.015
	Test-plan	97.578 ± 1.176	1.244 ± 0.150	0.065 ± 0.017
PTV2	Treat-plan	97.636 ± 1.152	1.131 ± 0.052	0.312 ± 0.029
	Test-plan	97.478 ± 1.247	1.135 ± 0.052	0.322 ± 0.025

**Table 4:** Normal organs evaluation in T4 patients.

		Dmin (Gy)	Dmax (Gy)	Dmean (Gy)
Brainstem	Treat-plan	14.443 ± 6.200	57.639 ± 4.098	34.802 ± 5.137
	Test-plan	13.972 ± 5.559	58.077 ± 4.113	35.279 ± 4.561
Spinal cord	Treat-plan	7.166 ± 7.823	37.952 ± 1.665	28.074 ± 4.114
	Test-plan	7.203 ± 7.858	38.104 ± 1.812	28.226 ± 4.078
Temporal Lobe-L	Treat-plan	3.965 ± 2.844	70.274 ± 4.007	23.380 ± 7.133
	Test-plan	4.464 ± 3.588	70.387 ± 4.181	24.009 ± 7.618
Temporal Lobe-R	Treat-plan	3.611 ± 2.412	68.992 ± 5.008	22.203 ± 5.537
	Test-plan	4.056 ± 3.050	69.176 ± 5.023	22.670 ± 6.014
optic chiasma	Treat-plan	39.904 ± 16.111	57.267 ± 12.110	49.924 ± 14.132
	Test-plan	40.848 ± 14.960	57.274 ± 12.002	50.492 ± 13.670

## Discussion

In the era of IMRT, the delineation of CTVp, which has remained a contentious topic, is one of the most crucial factors for achieving a good prognosis in NPC patients. In this study, we evaluated 222 radiotherapy plans and found that in T4 NPC patients, the more than 95% of PTV1 area could still be covered by the 60 Gy dose line even if CTVp1 was not delineated, which indicates that CTVp1 delineation in these patients is redundant. However, in T1–3 patients, if CTVp1 was omitted, just a few patients achieved a PTV1-TC of 95% or more, and even fewer in those who received induced chemotherapy. In addition, further analysis revealed that the TC of the original PTV1 area of the test plans was related to GTV.

In contrast to our findings, results from a study by Lin et al. (8) showed that the original PTV1 area can achieve ideal dose coverage without sketching the CTVp1 in NPC patients of all stages and not only in T4 patients. The discrepancy between this study and ours is mainly attributed to the non-conformity in the delineation of the target area. In the CTVp1-omitting test-plan, we evaluated the dose coverage of PTV1 that had already been created in the original treatment plan, whereas Lin et al. outlined a new CTVp1 for evaluation based on a smaller CTVp2. Based on their previous results that the maximum distance from GTVnx to CTVp2 in T4 patients (7.5 mm) is significantly shorter than T1–3 patients, but eventually led to the same recurrence pattern, Lin et al. used a uniform 8 mm margin from GTVnx to CTVp2 for all patients with varying stages. However, in practice, despite consistent general principles, there are specific differences in the delineation of the target area between each center (6, 13–16), and 8 mm is not the only criterion currently pursued. In our study, the treatment plans came from more than a dozen treatment groups, and provided that oncologists abided by the primary delineation principle. The outward expansion distance from GTVnx to CTV1 is not limited to a single value, as long as it is in the range of 5-10mm. Thus, our data is referable for most centers.

Previous studies (17, 18) have shown that IC reduces tumor volume, calling into question how the dose and target volume of gross tumor should be given. International guidelines for the delineation of CTVs for NPC recommend that the pre-induction volume should receive the full therapeutic dose regardless of post-IC shrinkage. However, a prospective study (19) reported that the post-IC tumor volume that received 70 Gy and pre-IC tumor volume that received at least 64 Gy did not reduce survival rates, and improved quality of life. The study by Wang et al. (20) also supported the delineation of GTV according to post-IC tumor volume. Because of these contradictory findings, we separately analyzed patients who received IC or not and discovered that IC did not affect the PTV1-TC of the test plan in T4 patients but significantly reduced the PTV1-TC compliance rate in T1–3 patients.

Without affecting the dose coverage of the PTV1 area, eliminating CTVp1 did not change any of the other dose parameters of PTVnx, PTVnd, PTV2, or exposure dose to normal organs. Thus, only one CTVp2 needs to be sketched in these patients with T4 NPC. This would simplify the work of radiation therapists who would ordinarily sketch two CTVs (21–23) and would theoretically reduce the radiation dose given to adjacent organs compared with sketching only one CTVp1 because of the difference in radiation dose given (i.e., 54 vs. 60 Gy) (24–26). However, this new approach for target delineation requires further verification using prospective clinical data.

In our test group, only a limited number of T1–3 nasopharyngeal carcinoma patients had satisfactory PTV1-TC. Although we are currently unable to accurately screen for these patients, our data showed that GTV was positively correlated with PTV1-TC, and thus, can be used to predict the compliance of PTV1-TC (AUC = 0.868, **Figure 4**), which may be a crucial factor for whether CTV1 needs to be outlined. Given that reduced CTV margins and radiation doses achieve long-term tumor control and mild late toxicity in early-stage NPC patients (27), the indications for removing CTVp1 may also be extended to more T1–3 NPC patients.

In conclusion, our study demonstrated the possibility of omitting CTVp1 in patients with T4 NPC according to dosimetric evaluations. However, for patients with T1–3 lesions, owing to the effects of IC and the difference in target delineation between clinicians, more cautious and individualized considerations are needed.

## Data availability statement

The original contributions presented in the study are included in the article/supplementary material. Further inquiries can be directed to the corresponding authors.

## Ethics statement

The studies involving human participants were reviewed and approved by the ethics committee of Jiangxi Provincial Hospital. The patients/participants provided their written informed consent to participate in this study.

## Author contributions

ZT and JL designed this study. XY made the target area plan. ZT and CY wrote this manuscript. SP, ZC, TL, QG, YQ, WX, FA, and GL collected and analyzed the data. All authors contributed to the article and approved the submitted version.

## Funding

This project was supported financially by grants from the Natural Science Foundation of China (81660453), Jiangxi Provincial Health Commission (20195440), and The Excellent Young Scientists Fund of Jiangxi Cancer Hospital (2021EYS03).

## Conflict of interest

The authors declare that the research was conducted in the absence of any commercial or financial relationships that could be construed as a potential conflict of interest.

## Publisher’s note

All claims expressed in this article are solely those of the authors and do not necessarily represent those of their affiliated organizations, or those of the publisher, the editors and the reviewers. Any product that may be evaluated in this article, or claim that may be made by its manufacturer, is not guaranteed or endorsed by the publisher.
